# Intentions and Attempts to Quit Smoking Among Sexual Minoritized Adult Smokers After Exposure to the Tips From Former Smokers Campaign

**DOI:** 10.1001/jamanetworkopen.2022.11060

**Published:** 2022-05-09

**Authors:** Yu Wang, Zongshuan Duan, Sherry L. Emery, Scott R. Weaver, Shannon R. Self-Brown, David L. Ashley, Jidong Huang

**Affiliations:** 1 Department of Health Policy and Behavioral Sciences, School of Public Health, Georgia State University, Atlanta; 2 Milken Institute School of Public Health, George Washington University, Washington, DC; 3 National Opinion Research Center at University of Chicago, Chicago, Illinois; 4 Department of Population Health Sciences, School of Public Health, Georgia State University, Atlanta

## Abstract

**Importance:**

Significant disparities exist in smoking behaviors by sexual minority status in the US.

**Objective:**

To examine potential differences in the associations between exposure to the Tips From Former Smokers (Tips) campaign and intentions and attempts to quit smoking by sexual minority status.

**Design, Setting, and Participants:**

This cross-sectional study used data from the wave 5 survey of the Population Assessment of Tobacco and Health study. Data from 8072 adults who were currently established cigarette smokers were collected from December 2018 to November 2019 and analyzed in August 2021. The Population Assessment of Tobacco and Health study is an ongoing cohort study representative of the noninstitutionalized US population. Sample weights were applied to account for the complex sampling strategies.

**Exposures:**

Dichotomized self-reported frequent Tips exposure (often and very often) and infrequent exposure (never, rarely, and sometimes).

**Main Outcomes and Measures:**

Outcomes were intention to quit within 12 months, any serious quit attempts in the past 12 months, and number of serious quit attempts in the past 12 months. Multivariate logistic and ordinal logistic regressions were used to estimate the weighted associations between exposure and each outcome. Interactions between Tips exposure and sexual minority status were examined to explore potential differences.

**Results:**

A total of 8072 participants (mean [SD] age, 44.7 [14.8] years; 3888 [53.2%] male; 4962 [67.4%] non-Hispanic White; and 915 [9.5%] sexual minoritized individuals [ie, those who identified as lesbian, gay, bisexual, or another minoritized sexual identity]) were included. Frequent Tips exposure was associated with higher odds of quit intentions and attempts overall (adjusted odds ratio [aOR], 1.25; 95% CI, 1.07-1.46 for intention to quit within 12 months; aOR, 1.26; 95% CI, 1.08-1.47 for serious quit attempts in the past 12 months; and aOR, 1.24; 95% CI, 1.06-1.44 for number of serious quit attempts in the past 12 months). These associations were significantly stronger for heterosexual smokers than sexual minoritized smokers, as indicated by the significant interaction terms (aOR, 0.58; 95% CI, 0.36-0.96 for intention to quit within 12 months; aOR, 0.41; 95% CI, 0.24-0.70 for serious quit attempts in the past 12 months; and aOR, 0.40; 95% CI, 0.24-0.67 for number of serious quit attempts in the past 12 months). Subgroup analysis showed that heterosexual smokers who reported frequent Tips exposure were more likely to intend to quit within 12 months (aOR, 1.29; 95% CI, 1.10-1.53), have had any serious quit attempts in the past 12 months (aOR, 1.34; 95% CI, 1.13-1.58), and have had more serious quit attempts (aOR, 1.32; 95% CI, 1.12-1.54) than heterosexual smokers who reported infrequent exposure. In contrast, there was no association for sexual minoritized smokers (aOR, 0.82; 95% CI, 0.52-1.30 for intention to quit within 12 months; aOR, 0.65; 95% CI, 0.39-1.07 for serious quit attempts in the past 12 months; and aOR, 0.62; 95% CI, 0.38-1.00 for number of serious quit attempts in the past 12 months).

**Conclusions and Relevance:**

These findings suggest that significant differences exist in the associations between Tips exposure and quit intentions or attempts by sexual minority status. More targeted campaign content for sexual minoritized smokers may be needed to increase quit intentions and attempts among this group.

## Key Points

**Question:**

Do smokers’ cessation behaviors after exposure to the Tips From Former Smokers (Tips) campaign differ by sexual minority status?

**Findings:**

In this cross-sectional study of the Population Assessment of Tobacco and Health study wave 5 data, among 8072 current established cigarette smokers, a positive association was found between frequent Tips exposure and intentions and attempts to quit smoking among those identifying as heterosexual but not among those who identified as lesbian, gay, bisexual, or another sexual identity.

**Meaning:**

More targeted campaign content may be needed to increase intentions and attempts to quit smoking among sexual minoritized smokers.

## Teaser

This cross-sectional study examines differences in intentions and attempts to quit smoking by sexual minority status among smokers exposed to the Tips From Former Smokers campaign.

## Introduction

In the US, cigarette smoking imposes a considerable burden on individuals, families, and society in terms of direct medical care cost and indirect cost in lost productivity.^1,2^ However, smoking-related burdens are not shared equally across population subgroups.^2^ In addition to geography, race and ethnicity, and socioeconomic status, recent evidence also suggests that important disparities exist by sexual minority status.^2,3,4^

Sexual minority status is defined based on an individual’s sexual orientation, which refers to “the sex of those to whom one is sexually and romantically attracted.”^5,6,7^ Categories of sexual minoritized populations typically include lesbian, gay, and bisexual.^5,6^ Some people identify as pansexual or queer in terms of sexual orientation.^6^ Sexual minoritized populations face significant discrimination and marginalization and experience related negative health outcomes, despite the substantial progress in social acceptance and civil rights in recent years in the US.^8,9^ Previous studies have indicated that psychological, normative, and environmental barriers may disproportionately affect sexual minoritized populations’ likelihood to quit tobacco successfully,^10,11^ and sexual minoritized populations were less likely to benefit from antitobacco interventions and services than the general population.^12,13,14^ The prevalence of current cigarette smoking among adult sexual minoritized populations was substantially higher than the corresponding prevalence among heterosexual people (19.2% vs 13.8% in 2019) in the US.^15^ A systematic review^3^ exploring the etiology of cigarette smoking disparities for sexual minoritized populations identified risk factors that were unique to them, such as internalized homophobia and reactions to disclosure of sexual orientation, and risk factors associated with smoking in the general population but with higher rates among sexual minoritized individuals, such as mental health issues and victimization. Targeted smoking cessation interventions are needed to reduce and eventually eliminate the disparities of smoking-related burdens among sexual minority populations.^3,16,17^

Mass media campaigns are among the most effective interventions to promote tobacco cessation.^18,19^ In 2012, the US Centers for Disease Control and Prevention (CDC) launched the first federally funded antismoking mass media education campaign: Tips From Former Smokers (Tips). The Tips campaign features compelling stories from real people living with serious diseases caused by smoking or secondhand smoke exposure and from people taking care of family members affected by smoking-related diseases or disabilities. The content of the Tips campaign was developed based on experiences from several countries and underwent rigorous testing.^20^ Previous studies^20,21,22,23,24,25,26,27^ have found that the Tips campaign is positively associated with quitline registrants, quit intentions and attempts, and smoking cessation. An estimated 16 million smokers have attempted to quit, and approximately 1 million people have successfully quit, because of the Tips campaign.^21^ Of importance, the Tips campaign has also been shown to be cost-effective.^28,29^

The design of the Tips advertiesments considered different demographic characteristics of population groups, including sexual and gender minoritized populations. Currently, there are 3 real stories of lesbian, gay, bisexual, and transgender (LGBT) people with smoking-related diseases and disabilities at the CDC Tips website: Brian’s story, Ellie’s story, and Rose’s story.^30^ The webpage for each story included the participant’s biography, videos, and stories and additional smoking cessation resources. In addition to the CDC’s website, these advertisements are also airing on national and cable television, online, and streaming radio.

Despite the potential significant impact of the Tips campaign on sexual minoritized smokers’ cessation-related outcomes, no studies to our knowledge have focused on how exposure to the Tips campaign is associated with smoking cessation behaviors among sexual minoritized smokers compared with heterosexual smokers. To address this critical research gap, this study used data from the most recent wave of the Population Assessment of Tobacco and Health (PATH) study to examine potential differences in the associations between Tips exposure and quit intentions and attempts by sexual minority status. We hypothesized that no significant differences would be found between these groups.

## Methods

Data for this cross-sectional study were from the restricted-use files of the wave 5 survey (December 2018 to November 2019) of the PATH study, a large-scale, ongoing, nationally representative cohort study. Details about the study design can be found elsewhere.^31,32^ The PATH study data collection was conducted by Westat and approved by the Westat Institutional Review Board. All participants provided written informed consent. The study population was current established cigarette smokers (N = 8072), defined as adults (≥18 years of age) who have smoked 100 or more cigarettes in their lifetime and currently smoke cigarettes every day or some days. Observations with missing values were less than 2% for univariate analysis and approximately 5% for regression analysis. Pairwise deletion was used to handle missing data. This secondary data analysis was exempted from institutional review board review because the data were deidentified. This study followed the Strengthening the Reporting of Observational Studies in Epidemiology (STROBE) reporting guideline.

The exposure variable was self-reported frequent Tips exposure in the past 12 months. In the PATH study, all respondents were shown the image of the Tips campaign logo and asked, “In the past 12 months, how often have you seen or heard of any advertisements on television, the internet, or radio with ‘A Tip From a Former Smoker’ slogan or theme?” Answer options included never, rarely, sometimes, often, and very often. Frequent Tips exposure was measured as dichotomized yes (often and very often) and no (never, rarely, and sometimes). Sensitivity analyses that measured Tips exposure as ever vs never being exposed were also conducted.

The outcomes were intention to quit within 12 months (yes/no), past 12-month serious quit attempt (yes/no), and past 12-month number of serious quit attempts (none, 1 time, 2-3 times, and ≥4 times). A serious quit attempt was defined as stopped smoking for 1 day or longer because of trying to quit. The potential effect modifier was respondents’ sexual minority status. All respondents were asked “Do you consider yourself to be…,” with responses options of “straight,” “lesbian or gay,” “bisexual,” and “something else.” Respondents who identified themselves as lesbian or gay, bisexual, or something else were coded as sexual minoritized populations.

Covariates were biological sex (male and female), age (18-24, 25-39, 40-54, and ≥55 years), race and ethnicity (Hispanic, non-Hispanic Black, non-Hispanic other, and non-Hispanic White), level of education (less than high school, high school graduate, some college or associate’s degree, and bachelor’s degree or above), past 12-month health professionals’ advise to quit, health insurance coverage, past 12-month e-cigarette use, past 12-month other tobacco use (cigar, hookah, pipe, and smokeless tobacco, including snus and dissolvable tobacco), and state-level cigarette tax (excise tax per pack measured in dollars) and smoke-free air policies (comprehensive or not). Information on race and ethnicity was derived from respondents’ answers to the PATH survey. Race and ethnicity were assessed in this study as a covariate because the literature has shown it to be associated with smoking cessation behaviors.^4^ State-level cigarette tax and smoke-free air policies were extracted from the CDC’s State Tobacco Activities Tracking and Evaluation System and merged into the PATH study data set using respondents’ residency states.^33^ States that banned smoking in workplaces, restaurants, and bars were coded as having comprehensive smoke-free air policies.

### Statistical Analysis

Data management and analysis were conducted in August 2021 using Stata software, version 16.1 (StataCorp LLC). Wave 5 single-wave weights were applied to account for the complex sampling design and generate nationally representative estimates. Bivariate associations between each outcome and Tips exposure, as well as bivariate associations between Tips exposure and covariates, were estimated. Multivariate logistic regressions were used to estimate the adjusted associations between Tips exposure and quit intentions and attempts. The assumption of proportional odds was checked, and multivariate ordinal logistic regression was used to estimate the adjusted association between Tips exposure and number of serious quit attempts. Interaction between Tips exposure and sexual minority status was added to each model to explore potential differences in the associations between Tips exposure and outcomes by sexual minority status. In addition, potential differences by other sociodemographic factors (sex, age, race and ethnicity, and educational level) were also examined using the same method. Subgroup analysis was conducted for any significant interaction effect detected. All statistical tests were 2-tailed, with significance level set at *P* < .05.

## Results

A total of 8072 participants (mean [SD] age, 44.7 [14.8] years; 3888 [53.2%] male, 4180 [46.8%] female, 1130 [13.0%] Hispanic, 1258 [13.9%] non-Hispanic Black, 580 [5.8%] non-Hispanic other, and 4962 [67.4%] non-Hispanic White, and 915 [9.5%] sexual minoritized individuals) were included. A total of 2741 participants (30.2%) had used e-cigarettes and 2818 (31.9%) had used other tobacco products in the past 12 months. A total of 1325 participants (16.5%) reported frequent Tips exposure in the past 12 months, 3678 (45.1%) reported no Tips exposure in the past 12 months, 4117 (52.6%) planned to quit within 12 months, 2295 (27.7%) made at least 1 serious quit attempt in the past 12 months, and 664 (8.4%) made serious quit attempts 4 times or more in the past 12 months (Table 1).

**Table 1.** Descriptive Characteristics of Current Established Cigarette Smokers at Population Assessment of Tobacco and Health Study Wave 5

Characteristic	No. of smokers^a^ (N = 8072)
Frequent past 12-mo Tips exposure	
Yes	1325 (16.5; 15.6-17.5)
Often	847 (10.9; 10.1-11.8)
Very often	478 (5.6; 5.0-6.2)
No	6701 (83.5; 82.5-84.4)
Never	3678 (45.1; 43.8-46.5)
Rarely	1187 (14.6; 13.7-15.5)
Sometimes	1827 (23.8; 22.6-24.9)
Sex	
Male	3888 (53.2; 51.9-54.5)
Female	4180 (46.8; 45.5-48.1)
Age group, y	
18-24	1082 (7.2; 6.7-7.7)
25-39	2857 (34.7; 33.5-36.0)
40-54	2046 (28.6; 27.4-29.9)
≥55	2087 (29.4; 28.2-30.6)
Race and ethnicity	
Hispanic	1130 (13.0; 12.1-13.9)
Non-Hispanic Black	1258 (13.9; 13.0-14.7)
Non-Hispanic White	4962 (67.4; 66.1-68.6)
Non-Hispanic other^b^	580 (5.8; 5.2-6.5)
Educational level	
Less than high school	2402 (28.2; 27.0-29.3)
High school graduate	2100 (29.8; 28.5-31.1)
Some college or associate’s degree	2697 (31.3; 30.1-32.5)
Bachelor’s degree or above	827 (10.8; 10.0-11.7)
Health care professionals advise to quit	
Yes	3171 (39.8; 38.6-41.1)
No	4875 (60.2; 58.9-61.4)
Health insurance coverage	
Yes	6183 (78.5; 77.4-79.5)
No	1818 (21.5; 20.5-22.6)
Sexual orientation	
Straight or heterosexual	7051 (90.5; 89.7-91.2)
Gay, lesbian, bisexual, or other	915 (9.5; 8.8-10.3)
Past 12-mo e-cigarette use	
Yes	2741 (30.2; 29.1-31.4)
No	5328 (69.8; 68.6-70.9)
Past 12-mo other tobacco use	
Yes	2818 (31.9; 30.7-33.1)
No	5229 (68.1; 66.9-69.3)
Comprehensive smoke-free air policy	
Yes	3818 (45.7; 44.4-47.0)
No	4254 (54.3; 53.0-55.6)
State cigarette tax	8072 (1.71; 1.68-1.75)
Intend to quit within 12 mo	
Yes	4117 (52.6; 51.3-54.0)
No	3720 (47.4; 46.0-48.7)
Serious quit attempt in the past 12 mo	
Yes	2295 (27.7; 26.5-28.9)
No	5752 (72.3; 71.1-73.5)
No. of serious quit attempts in the past 12 mo	
None	5752 (72.3; 71.1-73.5)
1	507 (6.0; 5.4-6.6)
2-3	1121 (13.3; 12.4-14.2)
≥4	664 (8.4; 7.7-9.2)

Abbreviation: Tips, Tips From Former Smokers.

^a^ Data in parentheses are presented as weighted percentage (95% CI), except for state cigarette tax, which is mean (95% CI).

^b^ Non-Hispanic other includes non-Hispanic American Indian or Alaska Native, Asian Indian, Chinese, Filipino, Japanese, Korean, Vietnamese, other Asian, Native Hawaiian, Guamanian or Chamorro, Samoan, and other Pacific Islanders.

Bivariate associations between Tips exposure and covariates (eTable 1 in the Supplement) showed that self-reported exposure to the Tips campaign was significantly different by sex, age, and race and ethnicity. Heterosexual smokers were more likely to report frequent Tips exposure than sexual minoritized smokers (n = 1182 [16.9%]; 95% CI, 15.9%-18.0% vs n = 130 [12.9%]; 95% CI, 10.6%-15.6%; *P* = .01).

Bivariate associations between each outcome and Tips exposure (Table 2) showed that current established smokers who reported frequent Tips exposure were more likely to have intention to quit within 12 months (759 [59.4%]; 95% CI, 56.1%-62.6% vs 3403 [51.5%]; 95% CI, 50.0%-53.0%; *P* < .001), have had any past 12-month serious quit attempt (444 [33.2%]; 95% CI, 30.3%-36.2% vs 1840 [26.7%]; 95% CI, 25.4%-28.0%; *P* < .001), and have had more past 12-month serious quit attempts (125 [10.1%]; 95% CI, 8.3%-12.2% vs 536 [8.1%]; 95% CI, 7.3%-8.9% for those with ≥4 attempts; *P* < .001).

**Table 2.** Bivariate Associations Between Tips Exposure and Each Outcome Among Current Established Cigarette Smokers, Population Assessment of Tobacco and Health Study Wave 5

Outcome	Frequent Tips exposure, No. (weighted %; 95% CI)		*P* value
	Yes (n = 1325)	No (n = 6701)	
Intend to quit within 12 mo			
Yes	759 (59.4; 56.1-62.6)	3403 (51.5; 50.0-53.0)	<.001
No	527 (40.6; 37.4-43.9)	3165 (48.5; 47.0-50.0)	
Serious quit attempt in the past 12 mo			
Yes	444 (33.2; 30.3-36.2)	1840 (26.7; 25.4-28.0)	<.001
No	881 (66.8; 63.8-69.7)	4839 (73.3; 72.0-74.6)	
No. of serious quit attempts in the past 12 mo			
None	881 (66.8; 63.8-69.7)	4839 (73.3; 72.0-74.6)	<.001
1	101 (7.9; 6.4-9.7)	405 (5.7; 5.1-6.3)	
2-3	218 (15.2; 13.2-17.6)	896 (13.0; 12.0-14.0)	
≥4	125 (10.1; 8.3-12.2)	536 (8.1; 7.3-8.9)	

Abbreviation: Tips, Tips From Former Smokers.

Consistent with bivariate associations, adjusted associations (Table 3) showed that respondents who reported frequent Tips exposure were more likely to have an intention to quit within 12 months (adjusted odds ratio [aOR], 1.25; 95% CI, 1.07-1.46), have had any past 12-month serious quit attempt (aOR, 1.26; 95% CI, 1.08-1.47), and have had more past 12-month serious quit attempts (aOR, 1.24; 95% CI, 1.06-1.44). In addition, non-Hispanic Black and Hispanic individuals were more likely to have quit intentions and attempts than non-Hispanic White individuals. Results from sensitivity analysis showed that self-reported ever Tips exposure was significantly associated with higher odds of quit intentions but not quit attempts compared with never exposure (eTable 2 in the Supplement).

**Table 3.** Results of Multivariate Regressions Between Tips Exposure and Smoking Cessation Behaviors, Population Assessment of Tobacco and Health Study Wave 5

Outcome	aOR (95% CI)		
	Intention to quit in 12 mo (n = 7562)	Serious quit attempt in the past 12 mo (n = 7697)	No. of serious quit attempts in the past 12 mo (n = 7695)
Frequent past 12-mo Tips exposure			
Yes	1.25 (1.07-1.46)	1.26 (1.08-1.47)	1.24 (1.06-1.44)
No	1 [Reference]	1 [Reference]	1 [Reference]
Sex			
Male	0.92 (0.81-1.03)	0.96 (0.84-1.09)	0.97 (0.85-1.10)
Female	1 [Reference]	1 [Reference]	1 [Reference]
Age group, y			
18-24	1.20 (0.98-1.47)	1.28 (1.03-1.59)	1.27 (1.03-1.56)
25-39	1.23 (1.05-1.43)	1.05 (0.89-1.24)	1.04 (0.88-1.22)
40-54	1.17 (1.00-1.37)	0.84 (0.71-0.99)	0.84 (0.71-1.00)
≥55	1 [Reference]	1 [Reference]	1 [Reference]
Race and ethnicity			
Hispanic	1.34 (1.11-1.60)	1.39 (1.13-1.70)	1.44 (1.17-1.78)
Non-Hispanic Black	1.99 (1.69-2.34)	1.40 (1.19-1.65)	1.43 (1.22-1.68)
Non-Hispanic White	1 [Reference]	1 [Reference]	1 [Reference]
Non-Hispanic other^a^	1.14 (0.89-1.47)	1.12 (0.85-1.47)	1.10 (0.84-1.43)
Educational level			
Less than high school	0.67 (0.55-0.82)	0.83 (0.67-1.03)	0.82 (0.66-1.01)
High school graduate	0.65 (0.53-0.80)	0.91 (0.73-1.13)	0.91 (0.73-1.13)
Some college or associate’s degree	0.85 (0.69-1.03)	0.96 (0.79-1.18)	0.95 (0.78-1.16)
Bachelor's degree or above	1 [Reference]	1 [Reference]	1 [Reference]
Sexual orientation			
Straight/heterosexual	1 [Reference]	1 [Reference]	1 [Reference]
Gay, lesbian, bisexual, or other	0.89 (0.73-1.07)	1.19 (0.96-1.46)	1.20 (0.98-1.47)
Health professionals advise to quit			
Yes	1.74 (1.55-1.97)	1.79 (1.57-2.03)	1.75 (1.54-1.99)
No	1 [Reference]	1 [Reference]	1 [Reference]
Health insurance coverage			
Yes	1.03 (0.89-1.18)	1.27 (1.08-1.49)	1.27 (1.08-1.50)
No	1 [Reference]	1 [Reference]	1 [Reference]
Past 12-mo e-cigarette use			
Yes	1.17 (1.03-1.33)	1.37 (1.19-1.58)	1.37 (1.19-1.56)
No	1 [Reference]	1 [Reference]	1 [Reference]
Past 12-mo other tobacco use			
Yes	0.73 (0.64-0.83)	0.95 (0.82-1.10)	0.94 (0.81-1.08)
No	1 [Reference]	1 [Reference]	1 [Reference]
State cigarette tax	1.01 (0.95-1.08)	1.03 (0.96-1.10)	1.02 (0.95-1.09)
Comprehensive smoke-free air policy			
Yes	1.20 (1.05-1.37)	1.05 (0.91-1.21)	1.08 (0.94-1.24)
No	1 [Reference]	1 [Reference]	1 [Reference]

Abbreviations: aOR, adjusted odds ratio; Tips, Tips From Former Smokers.

^a^ Non-Hispanic other includes non-Hispanic American Indian or Alaska Native, Asian Indian, Chinese, Filipino, Japanese, Korean, Vietnamese, other Asian, Native Hawaiian, Guamanian or Chamorro, Samoan, and other Pacific Islanders.

Models exploring potential differences between sexual minoritized and heterosexual smokers (with interaction term between Tips exposure and sexual minority status) (eTable 3 in the Supplement) found that the associations between self-reported frequent Tips exposure and quit intentions and attempts were significantly stronger for heterosexual smokers than sexual minoritized smokers, as indicated by the significant interaction term in each model (aOR, 0.58; 95% CI, 0.36-0.96 for intention to quit within 12 months; aOR, 0.41; 95% CI, 0.24-0.70 for serious quit attempts in the past 12 months; and aOR, 0.40; 95% CI, 0.24-0.67 for number of serious quit attempts in the past 12 months). Results from subgroup analysis showed that heterosexual smokers who reported frequent Tips exposure were more likely to intend to quit within 12 months (aOR, 1.29; 95% CI, 1.10-1.53), have had any past 12-month serious quit attempt (aOR, 1.34; 95% CI, 1.13-1.58), and have had more past 12-month serious quit attempts (aOR, 1.32; 95% CI, 1.12-1.54). In contrast, there wsa no association among sexual minoritized smokers (aOR, 0.82; 95% CI, 0.52-1.30 for intention to quit within 12 months; aOR, 0.65; 95% CI, 0.39-1.07 for any serious quit attempt in the past 12 months; and aOR, 0.62; 95% CI, 0.38-1.00 for number of serious quit attempts in the past 12 months). Interactions between Tips exposure and other sociodemographic factors were not statistically significant.

## Discussion

This study used nationally representative data to investigate the associations between self-reported frequent Tips exposure and quit intentions and attempts among current established cigarette smokers in the US and explored potential differences between sexual minoritized and heterosexual smokers. Our results showed that frequent Tips exposure was associated with increased odds of quit intentions and attempts overall. However, contrary to our initial hypothesis, there was no association among sexual minoritized smokers, an important finding not reported in previous studies.

Several potential explanations, based on both theoretical and empirical evidence, may explain these differences. First, it is possible that Tips advertisements featuring content that resonates with sexual minoritized smokers were not aired or aired less often on media channels that are more popular among these populations. One study^34^ showed that smokers with mental health problems who were exposed to the Rebecca advertisements (featuring a participant with mental health problems) more frequently had a greater likelihood of quit intentions or attempts. However, there were no associations among smokers with mental health problems who were exposed to other (non-Rebecca) Tips advertisements. Similarly, for sexual minoritized smokers who reported frequent Tips exposure, if the content of the advertisements did not sufficiently resonate with them, they may not be motivated to quit smoking. The PATH survey did not identify specific Tips advertisements that were seen by participants. Thus, we were not able to examine the specific association between exposure to Tips advertisements that feature sexual minoritized populations and quit intentions or attempts among sexual minoritized smokers.

Second, it is possible that the content of the Tips advertisements that feature sexual minoritized participants may not be sufficiently tailored to promote smoking cessation behaviors among sexual minoritized smokers. Currently, the primary content of the Tips advertisements targeted at LGBT groups still largely focuses on smoking-related diseases and disabilities but not on the social stressors and triggers of smoking specific to these groups and how to overcome these barriers to achieve smoking cessation.^30^ Previous studies^3,16,35^ have found that sexual minoritized smokers may have different smoking triggers than their heterosexual counterparts, including to cope with stress and/or depression that results from the social discrimination, stigmatization, and marginalization they experience. Achieving equity in smoking cessation requires a comprehensive consideration of multilevel challenges and barriers faced by these groups in accessing and benefiting from cessation services and interventions.

Third, the effect of antismoking campaign among sexual minoritized smokers may be offset by targeted marketing by the tobacco industry. Evidence shows that the social stressors experienced by sexual minoritized populations may be exploited by the targeted marketing of the tobacco industry.^36,37^ For example, several qualitative studies^17,38^ identified positive attitudes toward targeted tobacco marketing among sexual minoritized populations because of the tobacco industry’s explicit recognition of the social challenges facing the sexual minority communities. In addition, a previous study^39^ also found that LGBT smokers were more likely to be exposed to and share messages related to tobacco advertising and promotion on social media compared with their non-LGBT counterparts. Efforts to counter the targeted marketing by the tobacco industry are needed to help reduce the demand of tobacco products among sexual minoritized populations. Countermarketing measures should be guided by the World Health Organization Framework Convention on Tobacco Control, which called for a comprehensive ban of all tobacco advertising, promotion, and sponsorship.^40^

In addition to the differential associations between Tips exposure and smokers’ quit intentions and attempts by sexual minority status, several other important findings emerged from this study. First, we found that the reach of the Tips campaign was low in 2018-2019 based on the PATH study data. Previous studies^20,34^ conducted in 2012 and 2016 found that more than 75% of smokers were exposed to at least 1 Tips advertisement during the campaign. In the present study, nearly half (45.1%) of smokers had never been exposed to any Tips advertisements in the past 12 months during the period of late 2018 to 2019. Only 16.5% of smokers reported frequent exposure. Second, contrary to previous studies^41,42^ that found that smokers who were ever exposed to the Tips campaign were more likely to exhibit smoking cessation behaviors, our study results suggested that only frequent Tips exposure was positively associated with serious quit attempts. Previous studies^21,22,23,24,26,43,44^ that evaluated the effect of the Tips campaign mostly used market-level gross rating points to measure exposure to Tips advertisements. Although gross rating points are less likely to suffer from measurement error and recall bias than self-reported exposure, gross rating points measure area-level aggregated exposure and may not accurately capture the individual-level exposure. Third, our study found that non-Hispanic White smokers were less likely to have quit intentions or attempts, indicating that other interventions may be needed to promote smoking cessation for this group. Fourth, consistent with findings from previous studies,^4,22,45^ our study did not observe effect modification by sex, age, race and ethnicity, and educational level, suggesting that the Tips campaign is effective in promoting quit intentions and attempts among racial minority smokers and smokers with low socioeconomic status.

Our study results have important implications related to the Tips campaign. First, our study suggests that efforts are needed to increase the intensity of exposure to the Tips campaign among smokers to increase their quit intentions and attempts at the population level. Second, it might be beneficial for the Tips advertisements to incorporate more content that targets and resonates with sexual minoritized smokers. Third, the Tips advertisements targeted at sexual minoritized smokers may need to be aired more frequently through the channels that are more popular among those populations.

### Limitations

This study has some limitations. One major limitation is that this study did not include smoking cessation as an outcome for 2 reasons. First, contemporary smoking status and antismoking campaign exposure are jointly determined. Although the Tips campaign has the potential to promote cessation, current smokers are more likely to be exposed frequently because they are the targeted audience. Second, the inconsistent measures of Tips exposure across PATH study waves do not allow us to examine the association between baseline frequent Tips exposure and subsequent smoking cessation at follow-up waves. More longitudinal studies are needed to investigate the effect of antismoking campaigns on sustained smoking cessation. In addition, the sample size of sexual minoritized populations was small in this study. Future studies that oversample these populations are needed. Another limitation is that this study used self-reported data, which may introduce recall bias and social desirability bias. Furthermore, because of data availability, we could not identify the reasons why Tips exposure was differentially associated with smoking cessation behaviors for heterosexual and sexual minoritized smokers. More detailed data, especially qualitative data, are needed. This study treated sexual minoritized populations as a homogeneous group. However, previous studies^46,47^ have shown that use of tobacco products varies within lesbian, gay, and bisexual populations. Future studies are needed to examine these groups separately and should expand to include gender minoritized populations, such as the transgender population.

## Conclusions

In this cross-sectional study, the reach and exposure frequency of the Tips campaign was low among US adults who were current established smokers during our study period. Frequent Tips exposure was positively associated with quit intentions and attempts overall. However, no associations were seen among sexual minoritized smokers. Efforts are needed to increase the reach and exposure frequency of the Tips campaign. More targeted content for sexual minoritized smokers may be needed to increase their quit intentions and attempts.

## Notes

Supplement. **eTable 1.** Bivariate Associations Between Frequent Tips Exposure and Covariates Among Current Established Cigarette Smokers, PATH Study Wave 5 **eTable 2.** Results of Multivariate Regressions Between Ever Exposure to the Tips Campaign and Smoking Cessation Behaviors, PATH Study Wave 5 **eTable 3.** Results of Multivariate Regressions Between Frequent Tips Exposure and Smoking Cessation Behaviors, With Interaction Between Tips Exposure and Sexual Minority Status, PATH Study Wave 5
