# Supplementary material for: Intentions and Attempts to Quit Smoking Among Sexual Minoritized Adult Smokers After Exposure to the Tips From Former Smokers Campaign
Source: JAMA Netw Open. 2022 May 9;5(5):e2211060. doi: 10.1001/jamanetworkopen.2022.11060 (PMC9086838; doi:10.1001/jamanetworkopen.2022.11060)
Supplement: Supplement. — eTable 1. Bivariate Associations Between Frequent Tips Exposure and Covariates Among Current Established Cigarette Smokers, PATH Study Wave 5 eTable 2. Results of Multivariate Regressions Between Ever Exposure to the Tips Campaign and Smoking Cessation Behaviors, PATH Study Wave 5 eTable 3. Results of Multivariate Regressions Between Frequent Tips Exposure and Smoking Cessation Behaviors, With Interaction Between Tips Exposure and Sexual Minority Status, PATH Study Wave 5 [file jamanetwopen-e2211060-s001.pdf]

Supplementary Online Content

Wang Y, Duan Z, Emery SL, et al. Intentions and attempts to quit smoking among sexual minoritized adult smokers after exposure to the Tips From Former Smokers campaign. *JAMA Netw Open*. 2022;5(5):e2211060. doi:10.1001/jamanetworkopen.2022.11060

eTable 1. Bivariate Associations Between Frequent Tips Exposure and Covariates Among Current Established Cigarette Smokers, PATH Study Wave 5

eTable 2. Results of Multivariate Regressions Between Ever Exposure to the Tips Campaign and Smoking Cessation Behaviors, PATH Study Wave 5

eTable 3. Results of Multivariate Regressions Between Frequent Tips Exposure and Smoking Cessation Behaviors, With Interaction Between Tips Exposure and Sexual Minority Status, PATH Study Wave 5

This supplementary material has been provided by the authors to give readers additional information about their work.

© 2022 Wang Y et al. *JAMA Network Open.*

eTable 1. Bivariate associations between frequent *Tips* exposure and covariates among current established cigarette smokers, PATH Study Wave 5.

Covariates	Frequent *Tips* Exposure				p-value
	Yes (n=1,325)		No (n=6,701)		
	n (%/mean)	95% CI	n (%/mean)	95% CI	
Sex					
Male	511 (13.3)	12.1 - 14.6	3,348 (86.7)	85.4 - 87.9	<0.001
Female	814 (20.2)	18.8 - 21.8	3,349 (79.8)	78.2 - 81.2	
Age group					
18 - 24	139 (12.5)	10.4 - 14.9	935 (87.5)	85.1 - 89.6	<0.001
25 - 39	390 (13.2)	11.8 - 14.8	2,453 (86.8)	85.2 - 88.2	
40 - 54	380 (17.6)	15.8 - 19.6	1,654 (82.4)	80.4 - 84.2	
55 or above	416 (20.3)	18.3 - 22.4	1,659 (79.7)	77.6 - 81.7	
Race/ethnicity					
Hispanic	131 (11.5)	9.3 - 14.2	993 (88.5)	85.8 - 90.7	<0.001
Non-Hispanic Black	293 (23.9)	21.2 - 26.7	951 (76.1)	73.3 - 78.8	
Non-Hispanic Other	82 (11.9)	8.9 - 15.6	490 (88.1)	84.4 - 91.1	
Non-Hispanic White	801 (16.5)	15.4 - 17.8	4,144 (83.5)	82.2 - 84.6	
Education					
Less than high school	366 (15.4)	13.7 - 17.3	2,022 (84.6)	82.7 - 86.3	0.16
High school graduate	364 (18.1)	16.2 - 20.2	1,730 (81.9)	79.8 - 83.8	
Some college or associate degree	464 (16.5)	15.0 - 18.2	2,229 (83.5)	81.8 - 85.0	
Bachelor's degree or above	127 (15.1)	12.4 - 18.3	697 (84.9)	81.7 - 87.6	
Sexual orientation					
Straight/Heterosexual	1,182 (16.9)	15.9 - 18.0	5,851 (83.1)	82.0 - 84.1	0.01
Gay, Lesbian, Bisexual or Other	130 (12.9)	10.6 - 15.6	778 (87.1)	84.4 - 89.4	
Health professionals advise to quit					
Yes	681 (22.4)	20.7 - 24.1	2,484 (77.6)	75.9 - 79.3	<0.001
No	643 (12.6)	11.5 - 13.8	4,208 (87.4)	86.2 - 88.5	
Health insurance coverage					
Yes	1,072 (17.4)	16.3 - 18.6	5,095 (82.6)	81.4 - 83.7	<0.001
No	245 (13.4)	11.7 - 15.4	1,561 (86.6)	84.6 - 88.3	
Past-12-month e-cigarette use					
Yes	442 (16.4)	15.3 - 17.7	2,285 (83.6)	82.3 - 84.7	0.81
No	833 (16.7)	15.1 - 18.5	4,415 (83.3)	81.5 - 84.9	
Past-12-month other tobacco use					
Yes	456 (16.7)	15.5 – 18.0	2,342 (83.3)	82.0 - 84.5	0.54
No	865 (16.1)	14.5 - 17.8	4,341 (83.9)	82.2 - 85.5	
Comprehensive smoke-free air policy					
Yes	583 (15.4)	14.1 - 16.8	3,212 (84.6)	83.2 - 85.9	0.04
No	742 (17.5)	16.1 - 18.9	3,489 (82.5)	81.1 - 83.9	
State cigarette tax	1,325 (1.69)	1.62 - 1.76	6,701 (1.72)	1.68 - 1.75	0.57

© 2022 Wang Y et al. *JAMA Network Open.*

eTable 2. Results of multivariate regressions between ever exposure to the *Tips* campaign and smoking cessation behaviors, PATH Study Wave 5.

	Intention to quit in 12 months (n=7,562)		Past-12-month serious quit attempt (n=7,697)		Past-12-month times of serious quit attempt (n=7,695)	
	OR	95% CI	OR	95% CI	OR	95% CI
Past-12-month *Tips* exposure						
Ever	1.16	1.03 - 1.30	0.96	0.85 - 1.09	0.98	0.86 - 1.10
Never	Ref		Ref		Ref	
Sex						
Male	0.91	0.81 - 1.02	0.94	0.83 - 1.07	0.95	0.84 - 1.08
Female	Ref		Ref		Ref	
Age group						
18 - 24	1.20	0.98 - 1.47	1.25	1.01 - 1.56	1.25	1.01 - 1.54
25 - 39	1.23	1.06 - 1.44	1.04	0.88 - 1.22	1.03	0.87 - 1.20
40 - 54	1.17	1.00 - 1.37	0.83	0.70 - 0.99	0.84	0.71 - 1.00
55 or above	Ref		Ref		Ref	
Race/ethnicity						
Hispanic	1.35	1.13 - 1.62	1.37	1.12 - 1.68	1.43	1.16 - 1.76
Non-Hispanic Black	2.01	1.71 - 2.36	1.42	1.21 - 1.67	1.45	1.23 - 1.71
Non-Hispanic Other	1.15	0.89 - 1.48	1.11	0.84 - 1.45	1.09	0.84 - 1.42
Non-Hispanic White	Ref		Ref		Ref	
Education						
Less than high school	0.67	0.55 - 0.83	0.84	0.68 - 1.03	0.82	0.67 - 1.01
High school graduate	0.64	0.53 - 0.80	0.92	0.74 - 1.14	0.91	0.73 - 1.14
Some college or associate degree	0.84	0.69 - 1.03	0.97	0.79 - 1.19	0.95	0.78 - 1.17
Bachelor's degree or above	Ref		Ref		Ref	
Sexual orientation						
Straight/Heterosexual	Ref		Ref		Ref	
Gay, Lesbian, Bisexual or Other	0.89	0.73 - 1.07	1.17	0.95 - 1.44	1.19	0.97 - 1.46
Health professionals advise to quit						
Yes	1.75	1.55 - 1.98	1.82	1.60 - 2.08	1.78	1.57 - 2.03
No	Ref		Ref		Ref	
Health insurance coverage						
Yes	1.02	0.89 - 1.18	1.27	1.08 - 1.50	1.28	1.08 - 1.50
No	Ref		Ref		Ref	
Past-12-month e-cigarette use						
Yes	1.18	1.03 - 1.34	1.38	1.20 - 1.59	1.37	1.20 - 1.57
No	Ref		Ref		Ref	
Past-12-month other tobacco use						
Yes	0.73	0.64 - 0.84	0.95	0.83 - 1.10	0.94	0.82 - 1.08
No	Ref		Ref		Ref	
State cigarette tax	1.01	0.95 - 1.08	1.03	0.96 - 1.10	1.02	0.95 - 1.09
Comprehensive smoke-free air policy						
Yes	1.20	1.05 - 1.37	1.05	0.91 - 1.21	1.07	0.93 - 1.23
No	Ref		Ref		Ref	

© 2022 Wang Y et al. *JAMA Network Open.*

eTable 3. Results of multivariate regressions between frequent *Tips* exposure and smoking cessation behaviors, with interaction between *Tips* exposure and sexual minority status, PATH Study Wave 5.

	Intention to quit in 12 months (n=7,562)		Past-12-month serious quit attempt (n=7,697)		Past-12-month times of serious quit attempt (n=7,695)	
	**OR**	**95% CI**	**OR**	**95% CI**	**OR**	**95% CI**
Frequent past-12-month *Tips* exposure						
Yes	1.31	1.11 - 1.54	1.35	1.14 - 1.59	1.33	1.13 - 1.56
No	Ref		Ref		Ref	
Sexual orientation						
Straight/Heterosexual	Ref		Ref		Ref	
Gay, Lesbian, Bisexual or Other	0.95	0.77 - 1.17	1.33	1.06 - 1.66	1.35	1.09 - 1.68
Tips exposure * Sexual minority status						
Frequent * sexual minority	0.58	0.36 - 0.96	0.41	0.24 - 0.70	0.40	0.24 - 0.67
Others	Ref		Ref		Ref	
Sex						
Male	0.92	0.81 – 1.03	0.96	0.84 - 1.09	0.97	0.85 - 1.10
Female	Ref		Ref		Ref	
Age group						
18 - 24	1.20	0.98 - 1.47	1.28	1.03 - 1.59	1.27	1.03 - 1.57
25 - 39	1.23	1.05 - 1.44	1.06	0.90 - 1.25	1.04	0.89 - 1.23
40 - 54	1.17	1.00 - 1.37	0.84	0.71 - 0.99	0.85	0.71 - 1.00
55 or above	Ref		Ref		Ref	
Race/ethnicity						
Hispanic	1.33	1.11 - 1.60	1.38	1.13 - 1.70	1.44	1.17 - 1.78
Non-Hispanic Black	1.99	1.69 - 2.34	1.40	1.19 - 1.65	1.43	1.22 - 1.68
Non-Hispanic Other	1.15	0.89 - 1.47	1.13	0.86 - 1.48	1.10	0.85 - 1.44
Non-Hispanic White	Ref		Ref		Ref	
Education						
Less than high school	0.67	0.55 - 0.82	0.83	0.67 - 1.03	0.82	0.67 - 1.01
High school graduate	0.65	0.53 - 0.80	0.91	0.73 - 1.13	0.91	0.73 - 1.13
Some college or associate degree	0.85	0.69 - 1.03	0.96	0.79 - 1.18	0.95	0.78 - 1.16
Bachelor's degree or above	Ref		Ref		Ref	
Health professionals advise to quit						
Yes	1.74	1.54 - 1.97	1.78	1.57 - 2.03	1.75	1.54 - 1.99
No	Ref		Ref		Ref	
Health insurance coverage						
Yes	1.03	0.89 - 1.18	1.27	1.08 - 1.49	1.28	1.08 - 1.50
No	Ref		Ref		Ref	
Past-12-month e-cigarette use						
Yes	1.17	1.03 - 1.33	1.37	1.19 - 1.58	1.37	1.19 - 1.57
No	Ref		Ref		Ref	
Past-12-month other tobacco use						
Yes	0.74	0.65 - 0.84	0.96	0.83 - 1.11	0.94	0.82 - 1.09
No	Ref		Ref		Ref	
State cigarette tax	1.01	0.95 - 1.08	1.03	0.96 - 1.10	1.02	0.95 - 1.09
Comprehensive smoke-free air policy						
Yes	1.20	1.05 - 1.37	1.06	0.92 - 1.22	1.08	0.94 - 1.25
No	Ref		Ref		Ref	

© 2022 Wang Y et al. *JAMA Network Open.*
